# *N*-Glycosylation of the Discoidin Domain Receptor Is Required for Axon Regeneration in *Caenorhabditis elegans*

**DOI:** 10.1534/genetics.119.302492

**Published:** 2019-07-31

**Authors:** Tatsuhiro Shimizu, Yuka Kato, Yoshiki Sakai, Naoki Hisamoto, Kunihiro Matsumoto

**Affiliations:** Division of Biological Science, Graduate School of Science, Nagoya University, Chikusa-ku, 464-8602, Japan

**Keywords:** axon regeneration, *Caenorhabditis elegans*, *N*-glycosylation, DDR-2

## Abstract

Axon regeneration following neuronal injury is an important repair mechanism that is not well understood at present. In *Caenorhabditis elegans*, axon regeneration is regulated by DDR-2, a receptor tyrosine kinase (RTK) that contains a discoidin domain and modulates the Met-like SVH-2 RTK–JNK MAP kinase signaling pathway. Here, we describe the *svh-10*/*sqv-3* and *svh-11* genes, which encode components of a conserved glycosylation pathway, and show that they modulate axon regeneration in *C. elegans*. Overexpression of *svh-2*, but not of *ddr-2*, can suppress the axon regeneration defect observed in *svh-11* mutants, suggesting that SVH-11 functions between DDR-2 and SVH-2 in this glycosylation pathway. Furthermore, we found that DDR-2 is *N*-glycosylated at the Asn-141 residue located in its discoidin domain, and mutation of this residue caused an axon regeneration defect. These findings indicate that *N*-linked glycosylation plays an important role in axon regeneration in *C. elegans*.

AXON regeneration is necessary to restore the function of the nervous system following axon injury. This ability is governed by the interaction between the local extracellular environment and the intrinsic growth capacity of the neurons (Tedeschi and Bradke 2017). In adult mammals, regeneration following nerve damage occurs relatively efficiently in the peripheral nervous system, but less so in the central nervous system (Case and Tessier-Lavigne 2005; Tedeschi and Bradke 2017). This difference in the regeneration potential of the central *vs.* peripheral nervous system is due to differences in both extrinsic signals and intrinsic axon growth capabilities (Case and Tessier-Lavigne 2005). Of these, intrinsic regeneration signals are believed to predominate in determining regenerative success (He and Jin 2016; Mahar and Cavalli 2018). Accordingly, understanding the intrinsic mechanisms regulating axon regeneration may provide insights into possible treatments for neurological injury. Our understanding of these intrinsic signaling mechanisms remains limited at present.

The nematode *Caenorhabditis elegans* is an attractive model organism to investigate the mechanisms of injury-induced axon regeneration (Hammarlund and Jin 2014). Recent studies in this animal have identified biological pathways regulating axon regeneration. These pathways utilize signaling molecules that are well-conserved among species (Hisamoto and Matsumoto 2017). One is the JNK MAP kinase (MAPK) pathway, which consists of MLK-1 MAPKKK, MEK-1 MAPKK, and KGB-1 JNK. This pathway functions as a key intrinsic regulator of the initiation of regeneration in *C. elegans* (Nix *et al.* 2011). The JNK cascade is also downregulated by the MAPK phosphatase VHP-1, and *vhp-1* loss-of-function mutations cause hyperactivation of the JNK pathway (Mizuno *et al.* 2004). To identify new genes that function in the JNK pathway, we previously used an RNA interference screen to identify genetic suppressors of the *vhp-1* phenotype, and we termed these *svh* (suppressor of *vhp-1*) genes (Li *et al.* 2012). The *svh-1* gene encodes a growth factor-like protein homologous to mammalian HGF. The *svh-2* gene encodes a homolog of the mammalian Met, a receptor for HGF. The *C. elegans* SVH-2 acts as a receptor tyrosine kinase (RTK) that activates the JNK pathway via tyrosine phosphorylation of the MAPKKK MLK-1 (Figure 1A) (Li *et al.* 2012). The *svh-4* gene is identical to *ddr-2* and encodes an RTK homologous to the mammalian discoidin domain receptor (DDR), which is activated by collagen EMB-9 (Hisamoto *et al.* 2016). EMB-9–DDR-2 signaling regulates axon regeneration by modulating the SVH-1–SVH-2–JNK pathway (Figure 1A).

**Figure 1:**
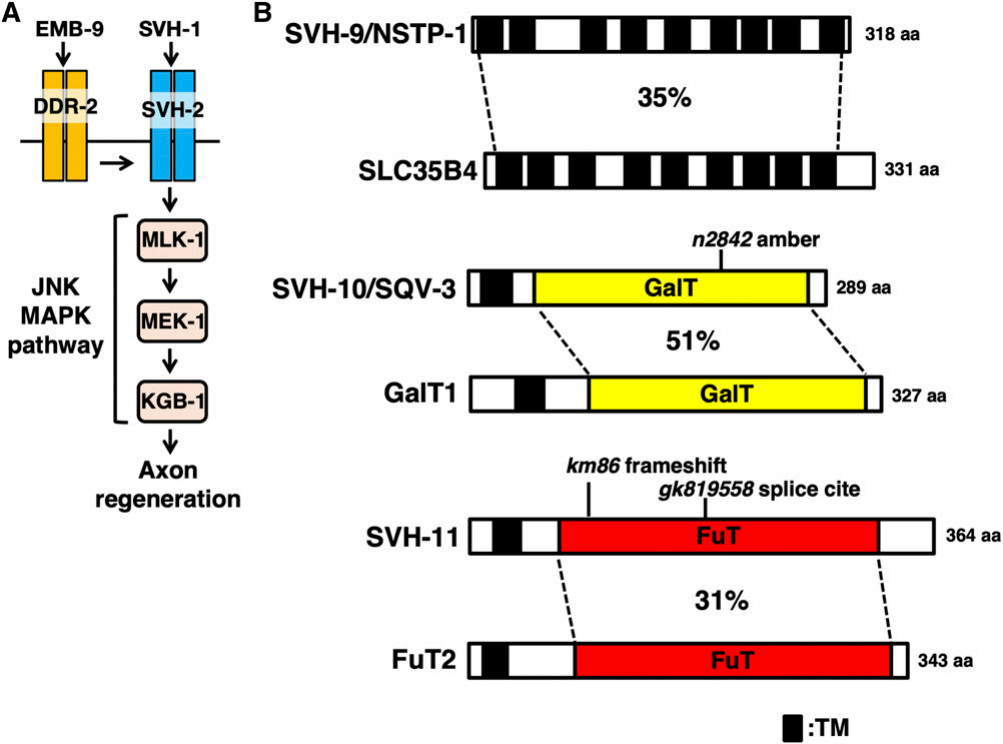
Identification of *svh-9/**nstp-1*, *svh-10*/*sqv-3*, and *svh-11* genes. (A) EMB-9 collagen–DDR-2 discoidin domain RTK modulates the SVH-1 growth factor–SVH-2 RTK–JNK pathway in *C. elegans*. (B) Structures of SVH-9/NSTP-1, SVH-10/SQV-3, and SVH-11. Schematic domain diagrams of *C. elegans* SVH-9/NSTP-1, SVH-10/SQV-3, SVH-11 and their human counterparts (SLC35B4, GalT1, and FuT2) are shown. Domains are shown as follows: a transmembrane domain (TM; black), a Gal transferase domain (GalT; yellow), and a Fuc transferase domain (FuT; red). The sites of each mutation are indicated.

Glycosylation is involved in many developmental and functional processes in the nervous system (Breen *et al.* 1998). For example, several glycoproteins, such as myelin-associated glycoprotein and chondroitin sulfate, inhibit axon regeneration in the central nervous system in mammals (Baldwin and Giger 2015). Furthermore, in zebrafish, the collagen-modifying glycosyltransferase LH3 is critical for regenerative growth and the guidance of axons through collagen type IV α5 (Isaacman-Beck *et al.* 2015). Glycosylation enzymes construct glycans in a sequential series of enzymatic steps (Schwarz and Aebi 2011). Glycosyltransferases mediate the transfer of a monosaccharide molecule from a nucleotide sugar donor substrate to a peptidic acceptor substrate.

In this study, we investigate the roles of SVH-10 and SVH-11 in regulating axon regeneration. These two gene products are involved in protein glycosylation, and genetic analysis suggests that SVH-11 functions between DDR-2 and SVH-2 in this glycosylation pathway. Furthermore, we show that DDR-2 is *N*-glycosylated at Asn-141 in its extracellular domain and that loss of *N*-glycosylation at this site decreases the signaling capability of DDR-2 in axon regeneration. These results indicate that *N*-linked glycosylation plays an important role in the control of *C. elegans* axon regeneration.

## Materials and Methods

### C. elegans strains

The *C. elegans* strains used in this study are listed in Supplemental Material, Table S1. All strains were maintained on nematode growth medium plates and fed with bacteria of the OP50 strain, as described previously (Brenner 1974). For heat shock treatment, worms on the nematode growth medium dishes were incubated at 37° for 30 min and then incubated at 20° for 4 hr.

### Plasmids

The Psqv-3::sqv-3 plasmid was generated by amplifying ∼2.3 kb of the DNA fragment from the N2 genome by PCR, using primer 1 (5′-gctcgttcttcaagagcgcatcagca-3′) and primer 2 (5′-gacgtcccgggactatgatttgcaataaggtgtcca-3′), and subcloning this into the pCR2.1-TOPO vector. Punc-25::sqv-3 was generated by inserting the *sqv-3* complementary DNA, which was amplified from a pACT *C. elegans* complementary DNA library (Sakamoto *et al.* 2005), using primer 3 (5′-tctagaatgaagctcaaaacgcggttaatctt-3′) and primer 2, into the pSC325 vector. Punc-25::*svh-11* was generated by amplifying the *svh-11*-coding DNA from N2 genome by PCR, using primer 4 (5′-gctagcatgcgattgtttcattttttgaaatttttgacaa-3′) and primer 5 (5′-ccatggtcagctcctcacaactgtaaaactattagcgc-3′), and inserting this into the pSC325 vector. Punc-25::ddr-2(N141A), Punc-25::ddr-2(N167A), Punc-25::ddr-2(N264A), and Punc-25::ddr-2(N353A) were generated by oligonucleotide-directed PCR, using Punc-25::ddr-2 (Hisamoto *et al.* 2016) as a template. Punc-25::ddr-2(N141A)::gfp and Punc-25::ddr-2(N141A; N167A)::gfp were generated by oligonucleotide-directed PCR, using Punc-25::ddr-2::gfp (Hisamoto *et al.* 2016) as a template. These variants were each confirmed by DNA sequencing. Phsp::ddr-2::gfp, Phsp::ddr-2(N141A)::gfp, and Phsp::ddr-2(N141A; N167A)::gfp were generated by inserting ddr-2::gfp, ddr-2(N141A)::gfp and ddr-2(N141A; N167A)::gfp DNA fragments derived from the Punc-25::ddr-2::gfp, Punc-25::ddr-2(N141A)::gfp, and Punc-25::ddr-2(N141A; N167A)::gfp plasmids, respectively, into the pPD49.78 vector. Phsp::ddr-2(K554E)::gfp plasmid was constructed by replacing a *Nru*I-*Bgl*II fragment of Phsp::ddr-2::gfp with the corresponding fragment in FLAG-Tpr-DDR-2C(K554E) (Hisamoto *et al.* 2016). The Pmyo-2::DsRed monomer plasmid and the Pmlk-1::mlk-1 fosmid have been previously described (Li *et al.* 2012).

### Transgenic animals

Transgenic animals were generated using a basic injection method (Mello *et al.* 1991). The Psqv-3::sqv-3, Punc-25::sqv-3, Punc-25::svh-11, Punc-25::ddr-2(N141A), Punc-25::ddr-2(N167A), Punc-25::ddr-2(N264A), Punc-25::ddr-2(N353A), Pmlk-1::mlk-1 fosmid, Punc-25::ddr-2::gfp, Punc-25::ddr-2(N141A)::gfp, Phsp::ddr-2::gfp, Phsp::ddr-2(N141A)::gfp, Phsp::ddr-2(K554E)::gfp, Phsp::ddr-2(N141A; N167A)::gfp, and Pmyo-2::DsRed monomer plasmids were used in *kmEx1271 [Psqv-3::sqv-3 (25 ng/μl)* + *Pmyo-2::DsRed monomer (25 ng/μl)]*, *kmEx1272 [Punc-25::sqv-3 (25 ng/μl)* + *Pmyo-2::DsRed monomer (25 ng/μl)]*, *kmEx1274 [Punc-25::svh-11 (25 ng/μl)* + *Pmyo-2::DsRed monomer (25 ng/μl)]*, *kmEx1281 [Punc-25::ddr-2(N141A)(25 ng/μl)* + *Pmyo-2::DsRed monomer (25 ng/μl)]*, *kmEx1282 [Punc-25::ddr-2(N167A)(25 ng/μl)* + *Pmyo-2::DsRed monomer (25 ng/μl)]*, *kmEx1283 [Punc-25::ddr-2(N264A)(25 ng/μl)* + *Pmyo-2::DsRed monomer (25 ng/μl)]*, *kmEx1284 [Punc-25::ddr-2(N353A)(25 ng/μl)* + *Pmyo-2::DsRed monomer (25 ng/μl)]*, *kmEx1287 [Pmlk-1::mlk-1(25 ng/μl)* + *Pmyo-2::DsRed monomer (25 ng/μl)]*, *kmEx1291 [Punc-25::ddr-2::gfp (25 ng/μl)* + *Pmyo-2::DsRed monomer (25 ng/μl)]*, *kmEx1292 [Punc-25::ddr-2(N141A)::gfp (25 ng/μl)* + *Pmyo-2::DsRed monomer (25 ng/μl)]*, *kmEx1293 [Phsp::ddr-2::gfp (25 ng/μl)* + *Pmyo-2::DsRed monomer (25 ng/μl)]*, *kmEx1295 [Phsp::ddr-2::gfp (50 ng/μl)* + *Pmyo-2::DsRed monomer (25 ng/μl)]*, *kmEx1294 [Phsp::ddr-2(N141A)::gfp (25 ng/μl)* + *Pmyo-2::DsRed monomer (25 ng/μl)]*, *kmEx1298 [Phsp::ddr-2(N141A)::gfp (50 ng/μl)* + *Pmyo-2::DsRed monomer (25 ng/μl)]*, *kmEx1299 [Phsp::ddr-2(K554E)::gfp (50 ng/μl)* + *Pmyo-2::DsRed monomer (25 ng/μl)]*, and *kmEx1300 [Phsp::ddr-2(N141A; N167A)::gfp (50 ng/μl)* + *Pmyo-2::DsRed monomer (25 ng/μl)]*, respectively. The *kmEx507* (Pmlk-1::mlk-1 fosmid), *kmEx1206* (Punc-25::svh-2), and *kmEx1202* (Punc-25::ddr-2) transgenes have been described previously (Li *et al.* 2012; Hisamoto *et al.* 2016).

### Generation of the *svh-11* mutation using CRISPR-Cas9

The *svh-11(km86)* deletion mutant was generated using the CRISPR-Cas9 system as described previously (Dokshin *et al.* 2018). The CRISPR RNA (5′-GCTTATCCAAGCATTTGAAC-3′) corresponding to a genomic sequence within the *svh-11* gene was synthesized (Integrated DNA Technologies: IDT) and co-injected with the trans-activating CRISPR RNA (IDT), *Streptococcus pyogenes* Cas9 3NLS (IDT) protein, and pRF4(*rol-6d*) plasmid into the KU501 strain. Each of the F1 animals carrying the transgene was transferred onto a new dish and used for single-worm PCR after egg laying to detect the presence of short insertions or deletions in the *svh-11* gene. The descendants of these animals were selected to obtain the *svh-11* homozygous mutant. The *svh-11(km86)* mutation is an 8-bp deletion in the *svh-11* gene, causing a frame shift and premature stop codon in exon 2.

### Axotomy

Laser microsurgery for determining axon regeneration was performed as described previously (Li *et al.* 2012). All animals were subjected to axotomy at the young adult stage. Imaged commissures that had growth cones or small branches present on the proximal fragment were counted as “regenerated.” Proximal fragments that showed no change after 24 hr were counted as “no regeneration.” A minimum of 13 individuals with one to three axotomized commissures were observed for most experiments.

### Microscopy

Standard fluorescence images of transgenic animals were observed under 60× and 100× objectives of a Nikon Eclipse E800 fluorescence microscope and photographed with a Hamamatsu ORCA 3CCD camera. The Olympus FV-500 and a Zeiss LSM 800 confocal microscope systems were also used for obtaining confocal fluorescence images.

### Biochemical experiments using worm extracts

Protein extraction from worms and immunoprecipitation were carried out as described previously (Hisamoto *et al.* 2019). For PNGase treatment, the bead pellets were further washed with wash buffer (20 mM Tris-HCl, pH 7.5, 50 mM NaCl, 5 mM EDTA) for several times and then treated with Rapid PNGase F (New England Biolabs) according to the instruction manual. The treated or untreated samples were boiled with SDS sample buffer and subjected to immunoblotting as described previously (Li *et al.* 2012; Hisamoto *et al.* 2018).

### Fucose blotting

Fucose was detected using *Aspergillus oryzae* lectin (AOL), specific for L-fucose, according to a previous report (Matsumura *et al.* 2007). Protein extracts fractionated by SDS–PAGE were electrophoretically transferred to a polyvinylidene difluoride membrane (Hybond-P; GE Healthcare). After blocking with 3% BSA in phosphate-buffered saline, the membrane was incubated with a 2 μg/ml solution of biotin-conjugated AOL (Tokyo Kasei) in TBST. After incubation for 1 hr at room temperature, the membrane was washed with Tris-buffered saline (TBS) and subsequently incubated with HRP-conjugated avidin (Thermo Fisher Scientific) diluted 1:2000 with TBS. After washing with TBS several times, the membrane was treated with an HRP chemiluminescent substrate reagent (Novex ECL; Invitrogen, Carlsbad, CA) and exposed to FUJI super RX Medical X-ray film (FUJIFILM) or images were obtained using FUSION FX (VILBER).

### Statistical analysis

The 95% confidence intervals were calculated by the modified Wald method and two-tailed *P* values were calculated using Fisher’s exact test, all of which were executed by using GraphPad software on the web (http://www.graphpad.com/quickcalcs/). A homology search was performed using the NCBI Protein BLAST program (https://blast.ncbi.nlm.nih.gov/Blast.cgi) with default parameters (blastp Algorithm, BLOSUM62 Matrix). Identification of conserved domains and alignments of amino acids were done using the programs on the NCBI BLAST (https://www.ncbi.nlm.nih.gov/Structure/cdd/wrpsb.cgi) and TMHMM Server web sites (http://www.cbs.dtu.dk/services/TMHMM-2.0/), as well as by the Genetyx-Mac program.

### Data availability

Strains and plasmids are available upon request. The authors affirm that all data necessary for confirming the conclusions of the article are present within the article, figures, and tables. Supplemental material available at FigShare: https://doi.org/10.25386/genetics.8797874.

## Results

### Protein glycosylation is involved in axon regeneration

We have previously isolated 92 *svh* genes involved in JNK signaling pathway (Li *et al.* 2012). In this study, we characterized three of these genes, *svh-9*, *svh-10*, and *svh-11*. The *svh-9* gene is identical to the *nstp-1* gene, which is homologous to the human solute carrier transporter 35 B4 gene (*SLC35B4*), encoding a Golgi apparatus-localized nucleotide sugar transporter with dual specificity for UDP-xylose (UDP-Xyl) and UDP-*N*-acetylglucosamine (UDP-GlcNAc) (Figure 1B, Figure S1A, and Figure S2). The *svh-10* gene has been previously identified as *sqv-3* (Herman *et al.* 1999), the protein product of which belongs to a family of glycosyltransferases including vertebrate β1,4-galactosyltransferases (GalT) that produce galactose-β1,4-GlcNAc linkages (Figure 1B, Figure S1A, and Figure S3) (Herman and Horvitz 1999; Bulik *et al.* 2000). The *svh-11* gene (Y5H2B.1 in Wormbase) encodes a protein homologous to the mammalian α1,2-fucosyltransferase 2 (FuT2), which transfers fucose in an α1,2 linkage to galactose (Figure 1B, Figure S1A, and Figure S4). Thus, SVH-9/NSTP-1, SVH-10/SQV-3, and SVH-11 are involved in protein *N*-glycosylation (Figure S1A).

**Figure 2:**
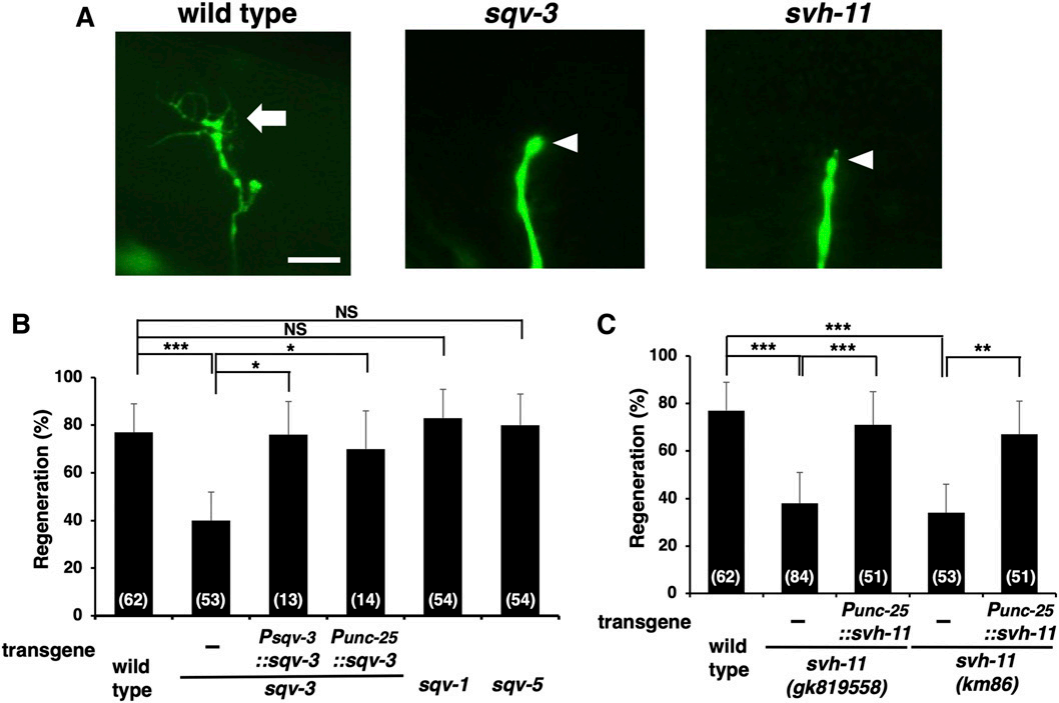
The *sqv-3* and *svh-11* genes are required for axon regeneration. (A) Representative D-type motor neurons in wild-type, *sqv-3*, and *svh-11* mutant animals 24 hr after laser surgery. In wild-type animals, a severed axon has regenerated a growth cone (arrow). In mutants, the proximal ends of axons failed to regenerate (arrowheads). Bar, 10 μm. (B and C) Percentages of axons that initiated regeneration 24 hr after laser surgery. The numbers (*n*) of axons examined are shown. Error bars indicate 95% confidence intervals. * *P* < 0.05, ** *P* < 0.01, *** *P* < 0.001 as determined by Fisher’s exact test. NS, not significant.

We first determined whether NSTP-1, SQV-3, and/or SVH-11 function in axon regeneration *in vivo*. Since the *nstp-1* loss-of-function mutation is as yet unavailable, we characterized regeneration in *sqv-3**(**n2842**)* and *svh-11(**gk819558**)* mutant animals. The *n2842* allele of the *sqv-3* gene contains an amber nonsense mutation, which results in a premature stop codon at Trp-184 (Figure 1B and Figure S3). The *svh-11(**gk819558**)* mutation occurs at an RNA splice site and generates a premature stop of the *svh-11* ORF (Figure 1B and Figure S4). Both *n2842* and *gk819558* alleles are probably null mutations. We subjected γ-aminobutyric acid–releasing D-type motor neurons to laser axotomy and monitored regeneration of their axons. These neurons extend axons from the ventral side to the dorsal side in the animal body (Figure 2A) (Nix *et al.* 2011). Axons severed by laser began to regenerate within 24 hr in wild-type animals at the young adult stage (Figure 2, A and B and Table S2). In contrast, the frequency of axon regeneration following laser axotomy was lower in *sqv-3** (**n2842**)* and *svh-11(**gk819558**)* mutants (Figure 2, A–C and Table S2). These results indicate that SQV-3 and SVH-11 are involved in axon regeneration after laser axotomy. In particular, their effects are injury-specific, since the *sqv-3** (**n2842**)* or *svh-11(**gk819558**)* mutation has no effect on normal nerve development (Figure S5).

To verify that the *sqv-3* mutation was responsible for this defect in axon regeneration, we generated the transgene *Psqv-3*::*sqv-3*, which contains the entire genomic *sqv-3* coding region and its promoter. Introduction of *Psqv-3*::*sqv-3* into *sqv-3** (**n2842**)* mutants rescued the defect in regeneration (Figure 2B and Table S2). We also found that expression of *sqv-3* in D-type neurons by the *unc-25* promoter rescued the axon regeneration defect in *sqv-3** (**n2842**)* mutants (Figure 2B and Table S2). Furthermore, similar to *sqv-3*, expression of *svh-11* in D-type neurons rescued the axon regeneration defect observed in *svh-11(**gk819558**)* mutants (Figure 2C and Table S2). To confirm the *svh-11* phenotype in axon regeneration, we used the CRIPSR/Cas9 system to generate the mutant *svh-11(km86)*, which encodes a frame shift and generates a premature stop of the *svh-11* ORF (Figure 1B and Figure S4). As observed in *svh-11(**gk819558**)* mutants, the *svh-11(km86)* mutant was also defective in axon regeneration, which was rescued by *Punc-25*::*svh-11* (Figure 2C and Table S2). These results thus demonstrate that SQV-3 and SVH-11 can act cell autonomously in damaged D-type neurons.

Glycosyltransferases can function in various glycosylation pathways, with *N*- and *O*-glycosylation being predominant (Figure S1, A and B). Previous studies have revealed that SQV-3 also participates in the biosynthesis of the *O*-linked glycosaminoglycans (Figure S1B) (Herman and Horvitz 1999; Herman *et al.* 1999; Bulik *et al.* 2000). SQV-1 is an UDP-glucuronic acid (UDP-GlcA) decarboxylase that catalyzes the conversion of UDP-GlcA to UDP-Xyl (Hwang and Horvitz 2002), which is then used to bind xylose to specific serine sites in proteins by the xylosyltransferase SQV-6 (Hwang *et al.* 2003b). Next, the SQV-3 protein transfers galactose from UDP-Gal to the serine-bound xylose, resulting in the biosynthesis of chondroitin and heparan sulfate glycosaminoglycans (Hwang *et al.* 2003a). However, in contrast to the *sqv-3* mutation, the *sqv-1**(**n2819**)* mutation had no effect on axon regeneration (Figure 2B and Table S2). The *n2819* allele replaces Pro-407 and Arg-410 with Leu and Gly, respectively. Furthermore, in *sqv-5**(**n3611**)* deletion mutants defective in chondroitin synthase (Figure S1B) (Hwang *et al.* 2003a), axons regenerate normally after axon injury (Figure 2B and Table S2). These results suggest that glycosaminoglycans are not required for axon regeneration. We therefore propose that the axon regeneration defect observed with the *sqv-3* and *svh-11* mutations is caused by a defect in *N*-glycosylation. Consistent with this, the *svh-11* mutation had no effect on vulval morphogenesis (Figure S6), whereas the *sqv-1*, *sqv-3*, *sqv-5*, and *sqv-6* genes are required for the vulval invagination and oocyte development (Herman *et al.* 1999).

### SVH-11 acts between DDR-2 and SVH-2 in the JNK pathway regulating axon regeneration

**Figure 3:**
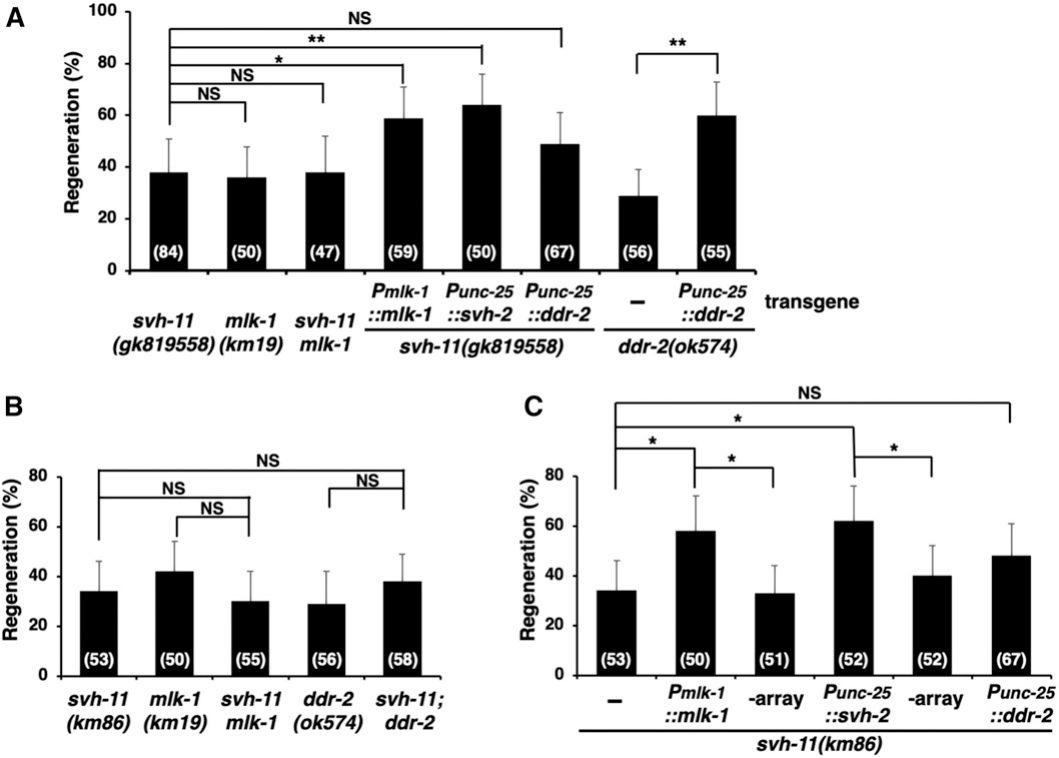
The relationship between SVH-11 and components involved in the JNK pathway in axon regeneration. (A–C) Percentages of axons that initiated regeneration 24 hr after laser surgery are shown. *svh-11(km86)* mutants carrying *Pmlk-1*::*mlk-1* or *Punc-25*::*svh-2* were analyzed for axon regeneration in nontransgenic siblings of the extrachromosomal transgenic lines (-array) (C). The numbers (*n*) of axons examined are shown. Error bars indicate 95% confidence intervals. * *P* < 0.05, ** *P* < 0.01 as determined by Fisher’s exact test. NS, not significant.

Our RNA interference screen for *svh* genes was designed to identify novel genes involved in the JNK pathway (Li *et al.* 2012). We therefore next investigated whether the effect of SVH-11 on axon regeneration involves the JNK pathway. MLK-1 acts as a MAPKKK in the JNK pathway (Figure 1A) (Mizuno *et al.* 2004). When we generated *svh-11(**gk819558**) **mlk-1**(**km19**)* and *svh-11(km86) **mlk-1**(*km19*)* double mutants, we found that axon regeneration in the double mutant was indistinguishable from that of either single mutant (Figure 3, A and B and Table S2). The *km19* allele of the *mlk-1* gene is a deletion mutation (Mizuno *et al.* 2004). This indicates that SVH-11 acts in the same pathway with MLK-1, and thus may function in the JNK pathway.

We next asked at what point SVH-11 acts in the JNK pathway during axon regeneration. Overexpression of *mlk-1* suppressed the regeneration defect observed in *svh-11(**gk819558**)* and *svh-11(km86)* mutants (Figure 3, A and C and Table S2), suggesting that SVH-11 functions upstream of MLK-1 in the JNK pathway. Activation of the JNK cascade following axonal injury is mediated by SVH-2, a homolog of the mammalian RTK Met, and its ligand SVH-1, an HGF-like growth factor. Activated SVH-2 in turn tyrosine phosphorylates MLK-1 (Li *et al.* 2012). In addition, DDR-2, another RTK that contains a discoidin domain, further modulates the SVH-1–SVH-2 pathway (Figure 1A) (Hisamoto *et al.* 2016). Since SVH-11 acts cell autonomously in axon regeneration (Figure 2C), a glycosylation target for SVH-11 should be produced in D-type motor neurons, excluding the possibility that SVH-1 might be the SVH-11 target. Furthermore, since SVH-11 functions upstream of MLK-1, the presumed glycosylation target could be SVH-2, DDR-2, or a protein acting proximal to these. We thus next examined if overexpression of *svh-2* or *ddr-2* using the *unc-25* promoter might reverse the defect in axon regeneration observed in *svh-11(**gk819558**)* and *svh-11(km86)* mutants. We found that overexpression of *svh-2* suppressed the regeneration defect in both *svh-11* mutants (Figure 3, A and C and Table S2). In contrast, although *Punc-25*::*ddr-2* was able to rescue the *ddr-2* defect, it failed to suppress the *svh-11* defect (Figure 3, A and C and Table S2). These results suggest that SVH-11 functions between SVH-2 and DDR-2 in the regulation of axon regeneration. We confirmed that SVH-11 functions in the same pathway with DDR-2, because the phenotype of *svh-11(km86)*; *ddr-2**(**ok574**)* double mutants was indistinguishable from that of either single mutant (Figure 3B and Table S2). The *ok574* deletion allele disrupts the kinase domain (Hisamoto *et al.* 2016).

### DDR-2 *N*-glycosylation is required for axon regeneration

**Figure 4:**
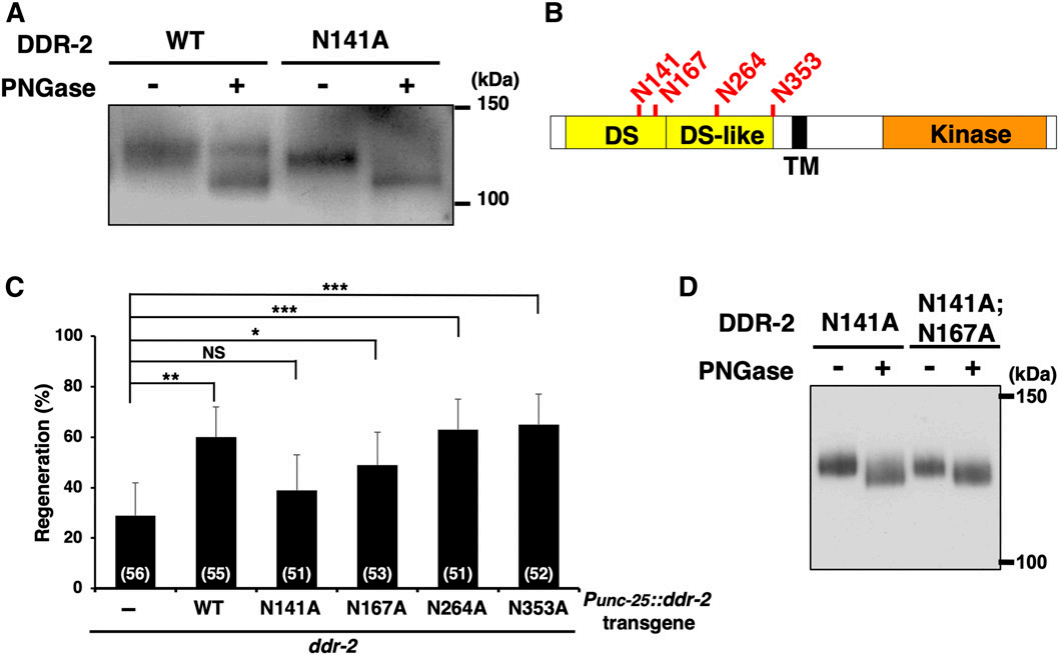
DDR-2 is *N*-glycosylated at Asn-141 in animals. (A) *N*-glycosylation of DDR-2. Wild-type animals carrying *Phsp*::*ddr-2*::*gfp* and *Phsp*::*ddr-2(N141A)*::*gfp* were subjected to heat shock at 37° for 30 min and incubated at 20° for additional 4 hr. Animal lysates were prepared and immunoprecipitated with anti-GFP antibody. The DDR-2::GFP immunoprecipitate was treated with (+) or without (−) PNGase, and immunoblotted with anti-GFP antibody. (B) Schematic diagram of DDR-2 domains. Potential *N*-glycosylation sites are indicated (red). Domains are shown as follows: discoidin (DS) and DS-like domains (yellow), a transmembrane domain (TM; black box), and a kinase domain (orange). (C) Effect of the *ddr-2(NA)* mutations on axon regeneration. Percentages of axons that initiated regeneration 24 hr after laser surgery are shown. The numbers (*n*) of animals examined are shown. Error bars indicate 95% confidence intervals. * *P* < 0.05, ** *P* < 0.01, *** *P* < 0.001 as determined by Fisher’s exact test. NS, not significant. (D) Effect of the *ddr-2(N141A*; *N167A)* double mutations on DDR-2 *N*-glycosylation. *ddr-2**(**tm797**)* mutants carrying *Phsp*::*ddr-2(N141A)*::*gfp* (N141A) or *Phsp*::*ddr-2(N141A*; *N167A)*::*gfp* (N141A; N167A) were subjected to heat shock at 37° for 30 min and incubated at 20° for additional 4 hr. Animal lysates were prepared and immunoprecipitated with anti-GFP antibody. The DDR-2::GFP immunoprecipitate was treated with (+) or without (−) PNGase, and immunoblotted with anti-GFP antibody.

To determine whether DDR-2 could be a direct target of *N*-glycosylation, we examined whether DDR-2 is *N*-glycosylated in animals. We expressed DDR-2::GFP fusion proteins from the heat shock promoter in wild-type animals. After heat shock treatment of the animals for 30 min and incubation for an additional 4 hr, animal lysates were prepared and immunoprecipitated with anti-GFP antibody. The DDR-2::GFP immunoprecipitate was divided in two aliquots, and one was treated with PNGase, an enzyme that cleaves all *N*-linked glycans (Tarentino *et al.* 1985). Immunoblotting with anti-GFP antibody identified a major band at ∼127 kDa, whereas the band in the PNGase-treated sample appeared at the lower molecular weight of ∼120 kDa, which would be expected for the deglycosylated DDR-2::GFP construct (Figure 4A). These results indicate that DDR-2 is *N*-glycosylated.

*N*-linked glycosylation occurs at Asn and can be predicted with high confidence by the presence of the motif Asn-Xxx-Ser/Thr (Schwarz and Aebi 2011). DDR-2 contains four such potential *N*-glycosylation sites in its extracellular region: Asn-141, Asn-167, Asn-264, and Asn-353 (Figure 4B). To address which of these sites are crucial for DDR-2 function in axon regeneration, we created a series of constructs, *ddr-2(N141A)*, *ddr-2(N167A)*, *ddr-2(N264A)*, and *ddr-2(N353A)*, which replace Asn with Ala and were expressed from the *unc-25* promoter. These were introduced into *ddr-2**(**ok574**)* mutants and axon regeneration was assessed. The DDR-2(N264A) and DDR-2(N353A) mutated forms were able to rescue the *ddr-2* defect in axon regeneration and thus were functional (Figure 4C and Table S2). The *ddr-2(N167A)* mutation also weakly rescued the *ddr-2* defect. In contrast, we found that the DDR-2(N141A) mutated form lost the ability to rescue the *ddr-2* defect (Figure 4C and Table S2. This indicates that Asn-141 in DDR-2 is important for its function in the control of axon regeneration.

To examine whether Asn-141 is *N*-glycosylated in animals, we expressed a DDR-2(N141A)::GFP fusion in wild-type animals and compared its molecular sizes to that of wild-type DDR-2::GFP. Western blot analysis showed that DDR-2(N141A)::GFP migrated faster than wild-type DDR-2::GFP and slower than PNGase-treated wild-type DDR-2::GFP (Figure 4A). Treatment of the DDR-2(N141A)::GFP sample with PNGase yielded a band of ∼120 kDa, similar to that of PNGase-treated wild-type DDR-2::GFP (Figure 4A). These results suggest that DDR-2 is *N*-glycosylated at Asn-141 and at least one other site. To test whether Asn-167 is an additional site for *N*-glycosylation, we expressed DDR-2(N141A)::GFP and DDR-2(N141A; N167A)::GFP double mutated form from the heat shock promoter in *ddr-2**(**tm797**)* mutants. We found that the position of DDR-2(N141A; N167A)::GFP on blots was similar to that of DDR-2(N141A)::GFP (Figure 4D). This indicates that DDR-2 is glycosylated on sites other than Asn-167.

### DDR-2 is fucosylated

**Figure 5:**
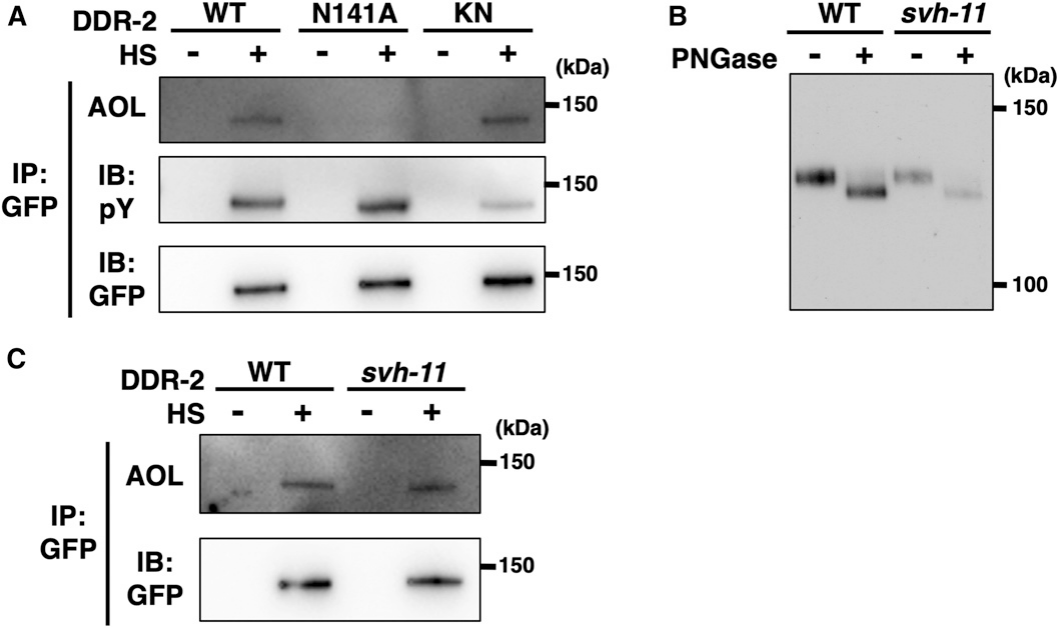
Fucosylation and tyrosine kinase activity of DDR-2. (A) Effects of the *ddr-2**(N141A)* mutation on fucosylation and tyrosine kinase activity of DDR-2. *ddr-2**(**tm797**)* mutants carrying *Phsp*::*ddr-2*::*gfp* (WT), *Phsp*::*ddr-2(N141A)*::*gfp* (N141A), or *Phsp*::*ddr-2(K554E)*::*gfp* (KN) were subjected to heat shock (+HS) at 37° for 30 min and incubated at 20° for additional 4 hr. Animal lysates were prepared and immunoprecipitated with anti-GFP antibody. The DDR-2::GFP immunoprecipitate was blotted with a lectin (AOL) specific for L-fucose and immunoblotted with anti–phospho-tyrosine (pY) and anti-GFP antibodies. Experiments were performed three times with similar results. (B) Effect of the *svh-11**(**gk819558**)* mutation on DDR-2 *N*-glycosylation. *ddr-2**(**tm797**)* (WT) and *svh-11(**gk819558**)*; *ddr-2**(**tm797**)* (*svh-11*) mutants carrying *Phsp*::*ddr-2*::*gfp* were subjected to heat shock at 37° for 30 min and incubated at 20° for additional 4 hr. Animal lysates were prepared and immunoprecipitated with anti-GFP antibody. The DDR-2::GFP immunoprecipitate was treated with (+) or without (−) PNGase, and immunoblotted with anti-GFP antibody. (C) Effect of the *svh-11**(km86)* mutation on DDR-2 fucosylation. *ddr-2**(**tm797**)* (WT) and *svh-11(km86)*; *ddr-2**(**tm797**)* (*svh-11*) mutants carrying *Phsp*::*ddr-2*::*gfp* were subjected to heat shock at 37° for 30 min and incubated at 20° for additional 4 hr. Animal lysates were prepared and immunoprecipitated with anti-GFP antibody. The DDR-2::GFP immunoprecipitate was blotted with a lectin (AOL) specific for L-fucose and immunoblotted with anti-GFP antibody.

The *svh-11* gene product is expected to transfer fucose in an α1,2 linkage to galactose (Figure S1A). We next examined whether DDR-2::GFP contains fucose. The expressed DDR-2::GFP proteins were analyzed by immunoprecipitation with anti-GFP antibody and immunoblotting with AOL, which specifically detects L-fucose (Matsumura *et al.* 2007). We found that DDR-2 proteins were fucosylated (Figure 5A), suggesting that *N*-glycosylated DDR-2::GFP indeed contains fucose. Furthermore, we investigated the effect of the *ddr-2(N141A)* mutation on DDR-2 fucosylation. We found that fucosylation was not detected in the DDR-2(N141A)::GFP mutated form (Figure 5A). These results suggest that the Asn-141 *N*-glycosylation site on DDR-2 is fucosylated.

We examined the effect of the *svh-11* mutation on the state of DDR-2 *N*-glycosylation. When DDR-2::GFP was expressed by the heat shock promoter in *svh-11(**gk819558**)*; *ddr-2**(**tm797**)* double mutants, the *svh-11* mutation did not affect the size of DDR-2::GFP proteins. When treated with PNGase, the band was converted to a lower molecular weight, similar to that observed in animals carrying the wild-type *svh-11* gene (Figure 5B). Furthermore, DDR-2::GFP was detected with AOL in *svh-11(km86)*; *ddr-2**(**tm797**)* mutants (Figure 5C). These results suggest that SVH-11 has no apparent effect on the DDR-2 fucosylation and that the other fucosyltransferases in *C. elegans* may be responsible for the modification.

### DDR-2 *N*-glycosylation is dispensable for its kinase activity in animals

We next investigated the contribution of *N*-glycosylation to DDR-2 function. Glycosylation is known to influence protein trafficking, therefore we investigated the localization of DDR-2::GFP protein in transgenic animals expressing *Punc-25*::*ddr-2*::*gfp*. Wild-type DDR-2::GFP showed a punctate localization pattern in D-type motor axons (Figure S7, A and B), consistent with our previous observations (Hisamoto *et al.* 2016). Similarly, the DDR-2(N141A)::GFP signals were localized in D-type motor axons (Figure S7, A and B). These results suggest that *N*-glycosylation of DDR-2 is not required for its efficient trafficking in axons.

The *N*-glycan acceptor site Asn-141 in *C. elegans* DDR-2 is located within its amino-terminal discoidin domain (Figure 4B). Since the discoidin domain is directly involved in collagen binding, *N*-glycosylation might affect collagen binding and consequently receptor activation. We therefore examined whether *N*-glycosylation of DDR-2 is required for activation of DDR-2 tyrosine kinase activity. Wild-type DDR-2::GFP or its variants were expressed in *ddr-2**(**tm797**)* mutants from the heat shock promoter, and the expressed DDR-2::GFP proteins were analyzed by immunoprecipitation with anti-GFP antibody and immunoblotting with anti–phospho-tyrosine (pY) antibody. We found that wild-type DDR-2::GFP was phosphorylated at tyrosine residues, whereas a kinase-negative mutant DDR-2(K554E)::GFP, in which Lys-554 was mutated to Glu (Hisamoto *et al.* 2016), exhibited weak tyrosine phosphorylation (Figure 5A). This suggests that DDR-2 autophosphorylates its tyrosine residues and that under normal conditions DDR-2 is thereby activated. Next, we tested the DDR-2(N141A) mutant and found that it retained tyrosine kinase activity similar to wild-type DDR-2 in animals (Figure 5A). Thus, the glycosyl moiety at the Asn-141 is dispensable for activation of DDR-2 by collagen in animals.

## Discussion

Glycosylation is involved in functional processes in the nervous system, such as axon regeneration in mammals and zebrafish (Breen *et al.* 1998; Isaacman-Beck *et al.* 2015). Edwards and Hammarlund recently reported that syndecan, a heparan sulfate proteoglycan, is required for growth cone function during axon regeneration in *C. elegans* (Edwards and Hammarlund 2014). In the absence of syndecan, regenerating growth cones are unstable and collapse. In this study, we show that axon regeneration in *C. elegans* appears to require *N*-linked glycosylation of DDR-2, a discoidin domain RTK that is activated by collagen. Indeed, we found that the Asn-141 residue in DDR-2 is modified by *N*-glycan and this modification contributes to its function in axon regeneration. These results demonstrate that *N*-linked DDR-2 glycosylation plays an important role in *C. elegans* axon regeneration.

The *N*-glycan acceptor site Asn-141 in *C. elegans* DDR-2 is located within the amino-terminal discoidin domain. Since the discoidin domain is directly involved in collagen binding, we asked whether the *N*-glycosylation of DDR-2 might affect the binding of collagen and consequently receptor activation. However, we observed this was not the case: the DDR-2(N141A) mutant retains the same tyrosine kinase activity as wild-type DDR-2 in animals. Thus, the glycosyl moiety at the Asn-141 is dispensable for activation of DDR-2 by collagen. Mammalian DDR proteins are also *N*-glycosylated (Curat *et al.* 2001). *N*-glycosylation at the Asn-211 residue within the discoidin-like domain of mammalian DDR1 controls its dimerization and autophosphorylation (Fu *et al.* 2014). There are differences between the *C. elegans* and mammalian DDR proteins, including differences in the positions of the *N*-glycan acceptor sites and the roles of *N*-glycan modification for DDR signaling. We have previously shown that in the *C. elegans* axon regeneration pathway, DDR-2 is necessary for the spatial accuracy of axon injury–induced activation of SVH-2, an Met-like RTK (Hisamoto *et al.* 2016). These data suggest that *N*-glycosylation of DDR-2 may be required for its association with SVH-2 to thereby restrict the localization of the SVH-1–SVH-2 signal to the JNK pathway. Thus, the role of Asn-141 *N*-glycosylation in the regulation of DDR-2 function revealed by these results will be followed by further experiments to understand the specific function and mechanism of *N*-glycosylation in DDR-2.

Glycosylation enzymes construct glycans in a sequential series of enzymatic steps. Glycosyltransferases mediate the transfer of a monosaccharide molecule from a nucleotide sugar donor substrate to a peptidic acceptor substrate (Schwarz and Aebi 2011). The SQV-3 protein catalyzes the addition of galactose-β1,4-GlcNAc linkages to proteins, and the SVH-11 protein catalyzes the addition of fucose to protein-linked galactose-β1,4-GlcNAc residues. One simple model is that the product of SQV-3 catalysis is subsequently used as the SVH-11 substrate. Fucose is a common monosaccharide component of cell surfaces and participates in many biological recognition events. Therefore, it is important to define the specificity of fucosyltransferases involved in fucosylation, potentially to target them for therapeutic purposes. The *C. elegans* genome contains at least 24 different genes encoding potential α1,2-fucosyltransferases and thus provides an attractive model system for exploring the potential roles of fucosylated glycans in animals. Because there is no effect of the *svh-11* mutation on DDR-2 fucosylation, it is likely that the SVH-11 target is not DDR-2, however, it is possible that one of the other 23 fucosyltransferases in *C. elegans* may be responsible for DDR-2 fucosylation. The SVH-9/NSTP-1 protein is homologous to mammalian SLC35, a protein that transports UDP-GlcNAc from the cytoplasm to the lumen of the Golgi apparatus (Ashikov *et al.* 2005). Therefore, NSTP-1 may act to translocate UDP-GlcNAc across Golgi membranes. SQV-3 and SVH-11 also appear to function in the lumen of the Golgi apparatus in the generation of proteoglycans. We propose that NSTP-1 transports UDP-GlcNAc, which may be used as substrates by SQV-3. Thus, these *svh-9*/*nstp-1*, *svh-10*/*sqv-3*, and *svh-11* genes encode components of a conserved glycosylation pathway that is required for axon regeneration.

In summary, we demonstrate that DDR-2 is *N*-glycosylated at Asn-141 in the discoidin domain. Therefore, our data shows that the *C. elegans* DDR-2 is one of a subgroup of RTKs that are critically regulated by *N*-glycosylation. *N*-glycosylation alters the function of DDR-2 in axon regeneration, thus highlighting the importance of this post-translational modification. These results provide a basis for further studies addressing the roles of DDR-2 glycosylation in the nervous system.
